# MAVTgsa: An R Package for Gene Set (Enrichment) Analysis

**DOI:** 10.1155/2014/346074

**Published:** 2014-07-03

**Authors:** Chih-Yi Chien, Ching-Wei Chang, Chen-An Tsai, James J. Chen

**Affiliations:** ^1^Community Medicine Research Center, Keelung Chang Gung Memorial Hospital, No. 200, Lane 208, Jijinyi Road, Anle District, Keelung 204, Taiwan; ^2^Division of Bioinformatics and Biostatistics, National Center for Toxicological Research, FDA, 3900 NCTR Road, HFT-20, Jefferson, AR 72079, USA; ^3^Department of Agronomy, National Taiwan University, No. 1, Section 4, Roosevelt Road, Taipei 106, Taiwan; ^4^Graduate Institute of Biostatistics and Biostatistics Center, China Medical University, Taichung, Taiwan

## Abstract

Gene set analysis methods aim to determine whether an a priori defined set of genes shows statistically significant difference in expression on either categorical or continuous outcomes. Although many methods for gene set analysis have been proposed, a systematic analysis tool for identification of different types of gene set significance modules has not been developed previously. This work presents an R package, called MAVTgsa, which includes three different methods for integrated gene set enrichment analysis. (1) The one-sided OLS (ordinary least squares) test detects coordinated changes of genes in gene set in one direction, either up- or downregulation. (2) The two-sided MANOVA (multivariate analysis variance) detects changes both up- and downregulation for studying two or more experimental conditions. (3) A random forests-based procedure is to identify gene sets that can accurately predict samples from different experimental conditions or are associated with the continuous phenotypes. MAVTgsa computes the *P* values and FDR (false discovery rate) *q*-value for all gene sets in the study. Furthermore, MAVTgsa provides several visualization outputs to support and interpret the enrichment results. This package is available online.

## 1. Introduction

DNA microarray technology enables simultaneous monitoring of the expression level of a large number of genes for a given experimental study. Much initial research on methods for data analysis has focused on the techniques to identify a list of differentially expressed genes. After selection of a list of differentially expressed gene, the list is then examined with biologically predefined gene sets to determine whether any sets are overrepresented in the list compared with the whole list ([1–3]). Mootha et al. [4] proposed gene set enrichment analysis (GSEA), which considers the entire distribution of a predefined gene set rather than a subset from the differential expression list. GSEA provides a direct approach to the analysis of gene sets of interest and the results are relatively easy to interpret. Furthermore, microarray experiments inherit various sources of biological and technical variability, and analysis of a gene set is expected to be more reproducible than an individual gene analysis.

GSEA is a statistical approach to determine whether a functionally related set of genes expresses differently (enrichment and/or deletion) under different experimental conditions. The GSEA approach has inspired the development of various statistical tests for identifying differentially expressed gene sets [5–16]. There are two fundamental hypotheses for GSEA: competitive hypothesis and self-contained hypothesis [8]. The competitive hypothesis tests if the association of a gene set with the phenotype is equal to those of the other gene sets. The self-contained hypothesis tests if the expression of a gene set differs by the experimental condition. In either test, resampling methods are typically used to generate the null distribution of test statistics. The null distributions of statistic under the competitive hypothesis are generated by gene sampling; the null distributions under the self-contained hypothesis are generated by subject-sampling. Various GSEA statistics have been proposed for testing either the competitive hypothesis or self-contained hypothesis. There are also hybrid-type methods that utilize gene and sample sampling (e.g., [5, 9]). The differences of the two hypotheses were evaluated and summarized in Nam and Kim [14] and Dinu et al. [15]. Gene sampling under the competitive hypothesis simply reassigns the genes into different gene sets. As a result, the sampled distributions are not independent. On the other hand, subject sampling is consistent with the null hypothesis, which the null distributions of statistical tests are identically and independently distributed. In addition, the self-contained hypothesis is a generalization from identification of differentially expressed genes to identification of differentially expressed gene sets. Therefore, we consider self-contained hypothesis and corresponding permutation *P* values in this paper.

In this paper, a statistical test to determine whether some functionally predefined classes of genes express differently under different experimental conditions is referred to as gene set analysis (GSA). When there are two experimental conditions, a GSA can be a one-sided or two-sided test. A one-sided test is to detect the changes of gene expressions in the gene set in one direction, either up- or downregulation. The two-sided test is to detect the changes in both up- and downregulation [6, 15]. When the goal is to detect coordinated changes in one direction, the one-sided hypothesis is appropriate. However, in an exploratory context, it is impossible to prespecify how individual genes in a gene set will respond in different conditions. Hence, two-sided hypothesis is generally suggested.

The original GSEA statistic was developed for a one-sided test to identify downregulated genes in type 2 diabetes mellitus subjects [4]. Other one-sided tests included T-like statistic [8]; MaxMean statistic [9]; standardized weighted sum statistic [10]; and OLS statistics [6]. Chen et al. [6] showed that OLS statistic performed well for GSA statistics in one-sided test. Two-sided tests were considered in most GSA statistics (e.g., [7, 11–17]). Tsai and Chen [16] proposed a multivariate analysis of variance (MANOVA) GSA test for two or more conditions. The MANOVA approach was compared with principal component analysis [13], SAM-GS [15], GSEA [5], MaxMean [9], analysis of covariance [7], and Goeman's global test [11]. They found that MANOVA test performed the best in terms of control of type I error and power.

The GSEA software only performs one-sided test in two conditions at a time [4]. Another software provides either one-sided or two-sided test; for example, BRB-Array tools [18] provided LS (KS) statistics, Goeman's global test, and MaxMean test. When the null hypothesis is that no genes in the gene set are differentially expressed, the two-sided is more appropriate than other methods.

When the purpose of study is to build up prediction rule based on gene expression profile, utilizing the existing biological knowledge, such as biological pathway or cellular function information, has been showed to improve the classification accuracy (e.g., [19–21]). The selection of predictors can be either preselected by testing or chosen based on classification algorithm, and the final identified predictors are also considered as differential expressed. Hsueh et al. [22] and Pang et al. [23] proposed a random forests-based differential analysis of gene set data in terms of predictive performance of gene sets. The analysis contained not only a classifier but also the feature importance of the input gene sets.

The gene set analysis has been considered to accommodate continuous phenotype. Linear combination test [17] has been extended from binary to continuous phenotype (LCT) by utilizing linear regression function [24]. Significance analysis of microarrays for gene sets (SAM-GS) [15] and global test [11] have also been extended in a generalized linear model (GLM) framework. Dinu et al. [24] compared the type I error rates and powers for the three methods and concluded that LCT approach is powerful and computationally attractive. The random forests-based approach could be also applied to continuous phenotype by modifying random forest classification to random forest regression. A small simulation experiment to compare the random forests-based and LCT is reported in this paper.

**Figure 1:**
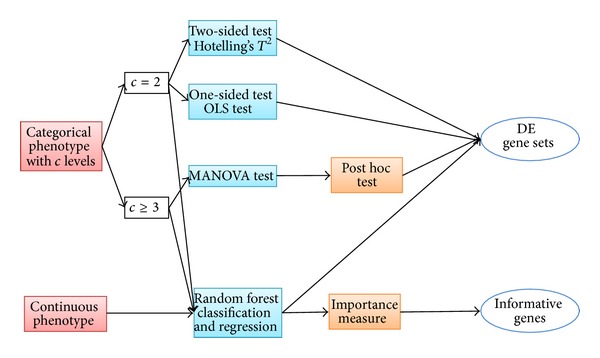
A schematic flowchart of GSEA using the MAVTgsa package.

The main purpose of this paper is to present the MAVTgsa R package tool for GSA for study with categorical phenotypes or continuous phenotypes. The OLS one-sided test, MANOVA test, and random forest-based analysis are implemented in MAVTgsa. When categorical phenotypes involve more than two classes, the three multiple comparison procedures are implemented: Dunnett, Tukey, and sequential pairwise comparison. The program provides two visualization plots: GSA plot, a *P* value plot of GSA for all gene sets in the study, and GST, a plot of the empirical distribution function of the ranked test statistics of a selected gene set. The researcher is able to summarize and visualize the gene expression data in gene set analysis. More importantly, the adjusted *P* value of family-wise error rate (FWER) [25] and FDR step-up procedure [26] are computed in this package. When the outcome of interest is a continuous phenotype, the random-forests based analysis is applied in the regression context.  Figure 1 describes the procedure of how to implement the MAVTgsa package for gene set enrichment analysis.

Section 2 describes the methods implemented in the MAVTgsa package. In Section 3, we present a description of the MAVTgsa package including data input, optional parameters, output, and result visualization. In Section 4, we apply to two real data sets, P53, and breast cancer, for illustration. This paper is concluded by a summary.

## 2. Methods

### 2.1. The OLS Test

The OLS test was developed to detect changes in one direction, either up- or downregulation, for a study with two experimental conditions. Consider a gene set consisting of **m** genes with two conditions of sample size **n**_1_ and **n**_2_. Let *y*_*ij*_ = (*y*_*ij*1_,…, *y*_*ijm*_) be the *m*-vector of intensities for simple *j*  (*j* = 1,…, *n*_*i*_) in *i*th condition (*i* = 1,2). Denote the standardized variable $y_{ijk}^{*}=(y_{ijk}-{\overset{-}{y}}_{k})/s_{k}$, where ${\overset{-}{y}}_{k}$ is the overall sample mean for the *k*th gene and *s*_*k*_ is the pooled standard deviation. Let *z*_*ik*_ = Σ_*j*_ *y*_ijk_*/*n*_*i*_ be the mean of the standardized variable for the *k*th in the *i*th condition. Denote the **z**_*i*_ = (*z*_*i*1_,…, *z*_*im*_) as the *m*-vector of the standardized mean variable *z*_*ij*_'s for the *i*th condition (*i* = 1,2). Let **d**_*i*_ be an *m*-dimensional vector for the mean difference between two conditions **d**_*i*_ = (**z**_1_ − **z**_2_). The O'Brien's OLS statistic [27] is

(1) $$\begin{array}{c} T_{\text{ols}}=\frac{1^{\prime }d_{i}}{{\left(1^{\prime }V_{i}1\right)}^{1/2}}, \end{array}$$

where 1 is a *m*_*i*_ × 1 vector of 1's and **V**_*i*_ is the pooled sample covariance matrix of **d**_*i*_. If **d**_*i*_ is a multivariate normal, then the OLS statistic *T*_ols_ has an approximately *t* distribution with *n*_1_ + *n*_2_ − 2 degrees of freedom.

The one-sided OLS statistic is the most widely used global test for the analysis of multiple clinical endpoints [27]. This test is very powerful when the changes in the gene expression are in the same direction. The direction of changes of significant gene set can be checked from the OLS statistic The OLS statistics account for gene set size and correlation structure [6]. In addition, the permutation *P* values for the OLS test are suggested to compute as the level of significance.

### 2.2. The MANOVA Test

Consider a gene set consisting of **m** genes with **c** conditions of sample size **n**_1_,…, **n**_**c**_. Let *y*_*ij*_ = (*y*_*ij*1_,…*y*_*ijm*_) be the *m*-vector of intensities for simple *j*  (*j* = 1,…, *n*_*i*_) in *i*th condition (*i* = 1,…, *c*). The MANOVA model [16] can be expressed as *y*_*ij*_ = *μ*_*i*_ + *e*_*ij*_, where *e*_*ij*_ is *m*-vector of residuals with Var(*e*_*ij*_) = Σ and *μ*_*i*_ is the *m*-vector of means for the *i*th condition. MAVTgsa occupied the Wilks' Λ as the statistic for MANOVA test. The formula of Wilks' Λ is given as

(2) $$\begin{array}{c} \Lambda =\Pi \frac{1}{\left(1+\lambda_{k}\right)}, \end{array}$$

where *λ*_*k*_'s are the eigenvalues of the matrix *S*  ( = *E* ^−1^ *H*), and *E* is within sum of squares matrix (sample covariance matrix) and *H* is between sum of squares matrix. The number of eigenvalues *k* is equal to the minimum of the number of genes (**m**) and the number of conditions minus 1  (**c** − 1). When the number of genes in the gene set is greater than the number of samples, the matrix *E* is singular and ill-conditioned. The shrinkage covariance matrix estimator (*s*_ij_*) proposed by Schafer and Strimmer [28] is applied to estimate the sample covariance matrix and given as

(3) $$\begin{array}{c} s_{ij}^{*}=\{\begin{array}{cc} s_{ii} & \text{if}\;\;i=j\\ r_{ij}^{*}\sqrt{s_{ii}s_{jj}} & \text{if}\;\;i\neq j \end{array} \end{array}$$

and $r_{ij}^{*}=r_{ij}\min\{1,\max\left(0,1-{\hat{\lambda }}^{*}\right)\}$, where *s*_*ii*_ and *r*_*ij*_, respectively, denote the empirical sample variance and sample correlation, and the optimal shrinkage intensity ${\hat{\lambda }}^{*}$ is estimated by

(4) $$\begin{array}{c} {\hat{\lambda }}^{*}=\frac{\sum_{i\neq j}\hat{\mathrm{Var}}\left(r_{ij}\right)}{\sum_{i\neq j}r_{ij}^{2}}. \end{array}$$

The distribution of Wilks' Λ under the null hypothesis of no difference in responses among the conditions was estimated by the permutation method. The Wilks' Λ test is equivalent to Hotelling's *T* ^2^ test when there are only two conditions. The Hotelling's *T* ^2^ statistic is

(5) $$\begin{array}{c} T^{2}=\frac{n_{1}n_{2}}{n_{1}+n_{2}}{\left({\overset{-}{x}}_{1}-{\overset{-}{x}}_{2}\right)}^{t}S_{p}^{-1}\left({\overset{-}{x}}_{1}-{\overset{-}{x}}_{2}\right), \end{array}$$

where ${\overset{-}{x}}_{i}$ and *S*_*i*_ denote the sample mean vector and sample covariance matrix of the *i*th group (*i* = 1,2), respectively, and *S*_*p*_ = ((*n*_1_ − 1)*S*_1_ + (*n*_2_ − 1)*S*_2_)/(*n*_1_ + *n*_2_ − 2) denotes the pooled covariance matrix. Using the shrinkage covariance matrix and permutation test, the *P* values of the Hotelling's *T* ^2^ test are computed. The *P* values of the MANOVA test are computed using the permutation method.

The MANOVA test is a multivariate generalization of the univariate analysis of variance (ANOVA) as Hotelling's *T* ^2^ test is the generalization of the univariate *t*-test. The parametric MANOVA and *T* ^2^ test statistics are commonly used for analyzing multivariate data. The MANOVA test uses a shrinkage covariance matrix estimator [28] to incorporate the correlations structure among the genes in the test statistic and account for the singularity and ill-condition of the sample covariance matrix. The MANOVA test uses the permutation method to compute the *P* values. Tsai and Chen [16] have compared the MANOVA test with several GSEA tests including the two one-sided tests: GSEA [5] and MaxMean [9], and four two-sided tests: ANCOVA [7], Global [11], PCA [13], and SAM-GS [15]. Their simulation showed that MANOVA test performs well in terms of controlling the type I error and power as compared to other tests, except that the ANCOVA [7] was more powerful when the variances were equal across all genes in the gene set.

### 2.3. Random-Forests Based Analysis

The random forests (RF) [29] is a popular classification and regression algorithm based on an ensemble of many classification/regression trees combined with a bootstrap sample out of the original dataset. The error rate of a classification tree or the mean of squared error (MSE) of a regression tree is calculated by the observations outside the bootstrap sample called the out-of-bag (OOB) data. Lower error rate and MSE indicates that the corresponding gene set is with higher prediction accuracy for a categorical phenotype and higher percent variance explained for a continuous phenotype, respectively. The variable selection for each split at the nodes of trees is conducted only from a small random subset of the predictors, so that there is no need to deal with the “small *n* large *p*” problem. A permutation based *P* value can be obtained as

(6) $$\begin{array}{c} P\text{-value}=\frac{\sum_{k=1}^{N}I\left\{R^{\left(k\right)}\leq R_{0}\right\}}{N}, \end{array}$$

where *R*_0_ is the observed score from the random forests analysis, *R* ^(*k*)^ is the score in the *k*th permutation, and *N* is the total number of permutations. The score is calculated as the OOB error rate and MSE for the categorical and continuous phenotypes, respectively. The gene set is considered as significantly expressed if the correspondent *P* value is less than or equal to the significance level *α*. To measure the importance of each predictor variable, the random forests algorithm assesses the importance of a variable by looking at how much prediction error increases or MSE decreases when that variable of the (OOB) data is permuted, while remaining variables are left unchanged. Here, the mean decrease in the Gini index (MDG) and the mean decrease in MSE are employed as the importance measures of genes for categorical and continuous phenotypes, respectively.

### 2.4. Multiple Comparisons Methods

In MANOVA test, the null hypothesis is rejected if one or more of the mean differences or some combination of mean differences among the genes in gene set differs from zero. If the null hypothesis is rejected, three multiple comparison procedures are implemented to identify which two conditions differ in expression between gene sets for studies with more than two conditions. Three multiple comparisons methods are Dunnett, Tukey, and sequential pairwise comparison. Dunnett test is specifically designed for situations where all groups are to be compared against the “reference” group. Tukey test is for all pairwise comparisons. When the conditions are in order, sequential pairwise comparison is appropriate to be occupied for comparing with previous condition.

## 3. MAVTGSA Package Description

MAVTgsa is a software tool to evaluate the expressions of a priori defined gene sets under different experimental conditions. The package implements essentially the method described in the previous section and the main functions are* MAVTn() *and* MAVTp()*.

### 3.1. MAVTn

For experiments with two or more than two conditions, the input parameters to perform the testing are described as follows.

*MAVTn(DATA, GS, Alpha, nbPerm, MCP)*

- * DATA* is a gene expression data matrix with samples in columns. The first row contains the information of the experimental condition of each sample. The remaining rows contain gene expression.
- * GS* is a binary matrix coded 0 or 1 with genes in rows. Each column represents a predefined gene set, with row equal to 1 indicating that the gene is in the gene set, and 0 otherwise.
- * Alpha* is the significance level.
- *nbPerm* specifies the number of permutations, and at least 5,000 is recommended.
- * MCP* specifies one of three multiple comparison methods, Dunnett = 1, Tukey = 2, and sequential pairwise comparison = 3. MCP can be ignored when the number of experimental conditions is 2.

The output of the* MAVTn()* function is a list of objects which contains the following.

(i) *P value:* a list of the gene sizes and the *P* values and adjusted *P* values of the statistical tests for GSA. The adjusted *P* values of the family-wise error rate (FWE) [25] and the FDR step-up procedure [26] are computed using permutation method. In case of two experimental conditions, the *P* values and adjusted *P* values for OLS and Hotelling's *T* ^2^ test are listed. In case of more than two conditions, the *P* values for MANOVA and* post hoc* tests are listed.
(ii) *Significant gene set:* a list of the *P* values of individual genes for those significant gene sets in statistical test of GSA.

In addition, a plot of *P* values for all gene sets is drawn to examine the adequacy of the assumptions on which the distributions of the test statistics are based.

Example of applying MAVTn to a simulated 3 conditions data is as follows:

 
*R> mu *<*− c(rep(1,5),rep(5,5))*
 
*R> Sigma *<*− diag(1,10)*
 
*R> set.seed (10) # fix random seed*
 
*R> GE *<*− rmvnorm (15,mu,Sigma) # generate 15 samples with 10 gene expression*
 
*R > set.seed(10) # fix random seed*
 
*R > GS *<*− matrix (1∗(runif (40,0,1)<=0.7),ncol=4,nrow=10) # simulate gene set matrix*
 
*R> cl *<*− c(rep(1,5),rep(2,5),rep(3,5)) #clinical treatment*
 
*R> data *<*− rbind(cl,t(GE))*
 
*R> MAVTn(data,GS,MCP = 1).*

### 3.2. MAVTp

For experiments with categorical or continuous phenotypes, the input parameters to perform the random forests analysis are described as follows.

*MAVTp(DATA, GS, nbPerm, Numoftree, Type, Impt)*

- * DATA* is a gene expression data matrix with samples in columns. The first row contains the information of the experimental condition of each sample. The remaining rows contain gene expression. If the first row is a factor, RF classification is assumed, otherwise RF regression is assumed.
- * GS* could be a binary matrix coded 0 or 1 with genes in rows. Each column represents a predefined gene set, with row equal to 1 indicating that the gene is in the gene set, and 0 otherwise.
- * nbPerm* specifies the number of permutations.
- * Numoftree* specifies the number of trees to grow.
- * Type* indicates categorical or continuous phenotype.
- * Impt* is an option for outputting the important measure.

The output of the* MAVTp()* function is a list of objects which contains the following.

- *P value:* a list of the *P* values of the random forests for GSA.
- * Important gene set:* a list of the importance measurement of individual genes for those significant gene sets.

Example of applying MAVTp to a simulated continuous phenotype data is as follows:

 
*R> mu *<*− c(rep(1,5),rep(5,5))*
 
*R> Sigma *<*− diag(1,10)*
 
*R> set.seed(15) # fix random seed*
 
*R> GE *<*− rmvnorm(15,mu,Sigma) # generate 15 samples with 10 gene expression*
 
*R> y = mvrnorm (1,rep(0,10),diag(rep(1,10))) # generate continuous phenotype*
 
*R> data *<*− rbind(y,GE)*
 
*R> GS *<*− matrix (1∗(runif (30,0,1)<=0.7),ncol = 3,nrow=10) # simulate gene set matrix*
 
*R> test_rf_con *<*− MAVTp(data,GS,nbPerm=1000, numoftree=500,type=“cont”,impt= TRUE).*

### 3.3. Visualization Plots

**Figure 2:**
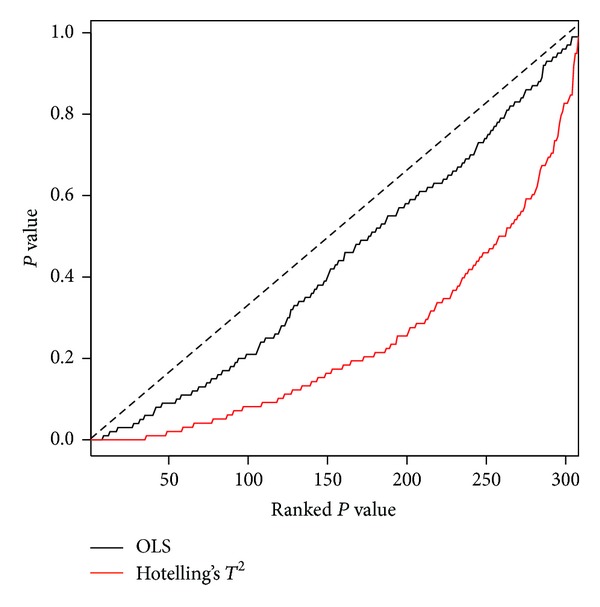
The GSA-plot for OLS test and Hotelling's *T* ^2^ test in P53 study. Both *P* values in OLS test (black line) and *T* ^2^ test (red line) are not close to the diagonal dash line. That means both tests could identify that several gene sets showed truly significant of the testing hypotheses.

**Figure 3:**
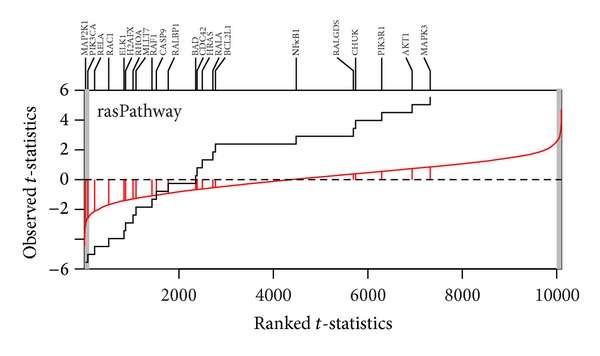
GST-plot for the gene set rasPathway in P53 dataset. The solid line is the empirical cumulative distribution function of the rank *t*-statistics for 10,100 genes in the array. The two-tailed shaded regions represent the *t*-statistics that had the *P* value less than 0.01. There are 22 tick marks above the plot which display the location of the *P* value of the genes from the gene set. The gene set shows underexpressed.

**Figure 4:**
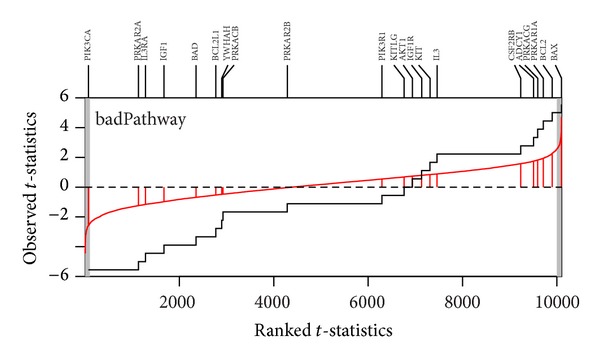
GST-plot for the gene set badPathway in P53 dataset. The solid line is the empirical cumulative distribution function of the rank *t*-statistics for 10,100 genes in the array. The two-tailed shaded regions represent the *t*-statistics that had the *P* value less than 0.01. There are 21 tick marks above the plot which display the location of the *P* value of the genes from the gene set. The gene set shows both under- and overexpressed.

**Figure 5:**
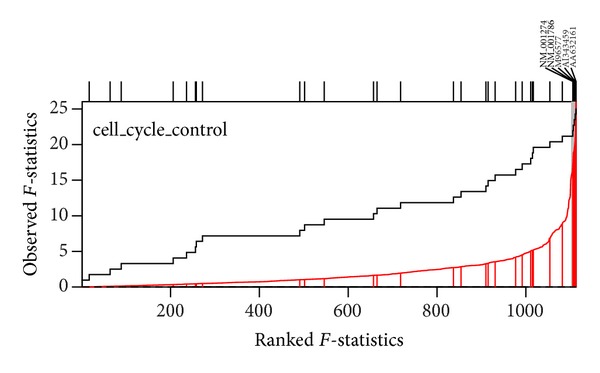
GST-plot for the gene set cell_cycle_control in breast cancer dataset. The solid line is the empirical cumulative distribution function of the rank *F*-statistics for 1,113 genes in the array. The shaded regions represent the *F*-statistics that had the *P* value less than 0.01. There are 5 tick marks above the plot which display the location of the *P* value of the genes from the gene set.

Two visualization plots are implemented,* GSA* and* GST*. The* GSA* plot (Figure 2) provides a *P* value plot of all gene sets considered in the study. The *P* value plot is the plot of ordered *P* value versus its rank. The *P* value plot can provide an overall assessment of differences in expression among conditions for all gene sets considered in the study. Under the null hypothesis of no differences, the *P* values should be uniformly distributed on the interval (0, 1); the *P* value plot should be a straight line. If a null hypothesis is not true, then its *P* value will tend to be small. The* GST* plot displays the relative direction (in two conditions) and statistics ranking for genes in a gene set. The* GST* plot is derived from the SAFE plot which provides the empirical distribution function for the ranked statistics of a given gene category [30]. The* GST* plot displays the ranked test statistics (red line) and empirical cumulative distribution function of these test statistics for expressed genes in a gene set (solid line). Tick marks above the plot display the location of genes with gene names. The shaded regions are set to represent the statistics that the *P* values below the given alpha value (Figures 3–5). The input parameters to perform the analysis are described as follows. Two examples are presented in next section to show the output of* GST *plot.

## 4. Results

### 4.1. P53 Study

The MAVTgsa was applied to a P53 dataset. The P53 dataset is from a study to identify targets of the transcription factor P53 from 10,100 gene expression profiles in the NCI-60 collection of cancer cell lines. There are 308 gene sets in the P53 study. The mutation status of the P53 gene has been reported for 50 cell lines included 17 wild-type and 33 mutation samples. The dataset is publicly available at the GSEA website (http://www.broad.mit.edu/gsea/).  Table 1 shows the result of fifteen gene sets in which the *P* values for OLS test are the top fifteen smallest *P* values under 10,000 permutations (*nbPerm* = 10,000). The significant level is set as 0.01 (*alpha* = 0.01). The *P* values for OLS test and MANOVA (Hotelling's *T* ^2^) were calculated and listed the *P* values of individual genes for those significant gene sets. The *P* values from the OLS test are different from the *P* values from two-sided *T* ^2^. The gene set rasPathway, HTERT_DOWN, and ngfPathway are highly significant (*P* < 0.01) by OLS test, but they are not significant in Hotelling's *T* ^2^ test. In contrary, the gene set badPathway is highly significant in Hotelling's *T* ^2^ test but not in OLS test. Figure 2 is the GSA-plot for two groups. Figures 3 and 4 are the GST plots for the gene set rasPathway and badPathway, respectively. In Figure 3, two of 22 genes are underexpressed with the *P* value less than 0.01. On the other hand, one of the 21 genes in badPathway shows underexpressed and one shows overexpressed in Figure 4. The results indicate that the power of methods to detect differential expressed gene set depends on the global pattern of genes within gene set. Combining the information from the two tests and GSA-plot will be useful to get biological meaningful interpretation.

### 4.2. Breast Cancer Dataset

We applied the MAVTgsa to a breast cancer dataset [31] and illustrate the RF, MANOVA, and a multiple comparison analysis. The dataset consisted of three conditions with 1,113 genes and 96 samples. Three conditions were tumor grades 1, 2, and 3 with the sample sizes of 11, 25, and 60, respectively. There were nine cancer related pathways for gene set analysis.  Table 2 shows the *P* values of the MANOVA and the post hoc Dunnett's analysis for each of the nine pathways.  Figure 5 is the GST plots for the 31 genes in the gene set cell_cycle_control. Five of the 31 genes are differential expressed with *P* value less than 0.01 in the breast cancer samples. In this analysis the total computation time used to perform the analysis was approximately four hours and 10 minutes with nbPerm = 10,000. A classification rule to classify the three tumor grades is also constructed. The error rates of 10-fold cross-validation are given in Table 2.

### 4.3. Simulation Study

**Figure 6:**
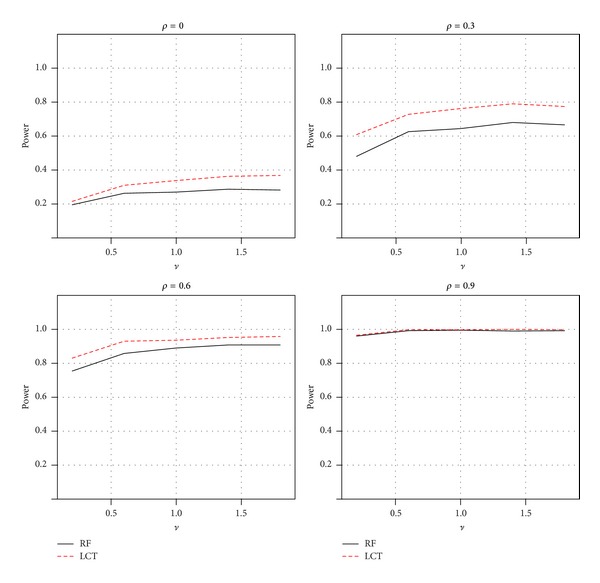
Power comparisons of two GSA methods for a linear association between gene sets and continuous phenotypes: random forests and LCT.

In order to understand how well the random forests-based analysis performs for continuous phenotypes, we conducted a number of simulations to evaluate the performance in terms of type I error and power and compare to the LCT method [24]. The simulation design was similar to that considered by Dinu et al. [24]. For each gene set of size *p* 24, we generated a *p* by *n* gene expression matrix *X*_*n*×*p*_ with sample size of *n* and a linear model was used to simulate the phenotype data associated with the gene set *X*. The gene expressions matrix *X* was generated from a multivariate normal distribution with mean from a uniform (0, 10) distribution, variance from a uniform (1, 5) distribution, and a mixed intragene set correlation structure as follows:

(7) $$\begin{array}{c} \rho_{ij}=\{\begin{array}{cc} \rho , & 1\leq i\neq j\leq p_{1};\\ \rho^{\left|i-j\right|}, & p_{1}+1\leq i\neq j\leq 2p_{1};\\ 0, & \text{otherwise}, \end{array} \end{array}$$

where the correlation *ρ* was set at 0, 0.3, 0.5, and 0.9 in this study. For each gene set, a continuous phenotype vector *Y*_*n*×1_ is generated from a multivariate normal distribution MVN(*Xμ*, **I**), where *μ* is the effect size vector with length *p* and **I** the identity covariance matrix. In the null model, with no association of gene expressions on the phenotype, we set *μ* to be 0 to investigate the type I error and the simulation scenarios varied according to sample sizes (*n* = 10, 20, or 50), gene set sizes (*p* = 20, 100, or 200), and the levels of correlation among genes (*ρ* = 0, 0.3, 0.5, or 0.9) within a gene set at different number of genes (*p*_1_ = 5, 20, or 40). In the alternative model, the sample size and gene set size were, respectively, fixed at 20 and 100, as well as only 10 of genes were simulated to be associated with the phenotype to investigate power of two gene set analysis methods. First, we randomly generated 5 of the first 20 components of *μ* from normal distribution *N*(*ν*, | *ν*|) and another five of the next 20 components of *μ* from normal distribution *N*(−*ν*, |*ν*|) as up- and downregulated genes, respectively. The rest of components of *μ* were set to be 0. The effect sizes (*ν*) for the associations were set at 0.2, 0.6, 1.0, 1.4, and 1.8. For each scenario, the simulation data were replicated 1000 times to estimate type I error rate or power. The *P* values were based on 1000 permutations. Power was then estimated as the proportion of significance using the nominal level of 0.05.  Table 3 showed the empirical type I errors using the nominal level of 0.05 for each scenario. The type I errors from the random forests method were reasonably close to or below the nominal level, while the LCT method appeared to have an inflated type I error rate in most cases. It indicates that the RF method gives a conservative conclusion. Such conservativeness may lead to power loss in detecting a difference.  Figure 6 illustrated the empirical powers using the nominal level of 0.05 for *ρ* = 0.0, 0.3, 0.6, and 0.9. As expected, the RF method was slightly inferior to the LCT method, while LCT was unable to adequately control the type I error rate. However, with increasing of correlations among genes, both methods appeared to be equivalent. The powers of both methods increased gradually with increasing correlations.

**Figure 7:**
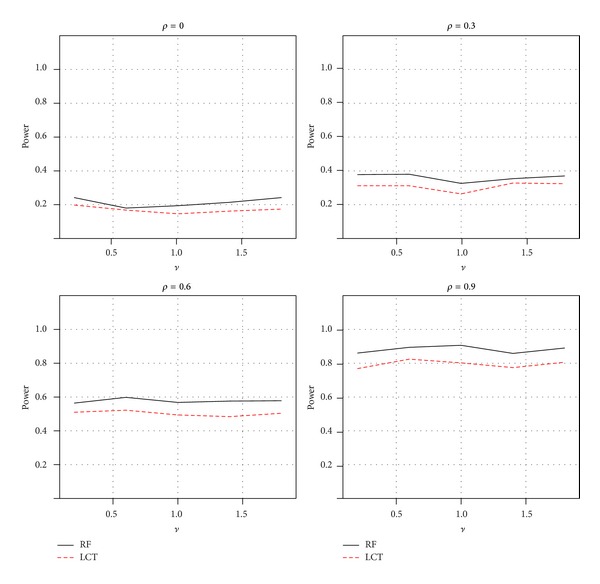
Power comparisons of two GSA methods for a nonlinear association between gene sets and continuous phenotypes: random forests and LCT.

In addition, to explore the robustness of the RF method with regard to nonlinear association data, the continuous phenotype vector *Y*_*n*×1_ was generated from a multivariate normal distribution MVN(exp⁡(*Xμ*), **I**).  Figure 7 showed the average power over 1000 simulations for each method using the nominal level of 0.05. As a result, the RF provided a more powerful test than the LCT method to detect the non-linear association between gene sets and continuous phenotypes.

## 5. Conclusion

The MAVTgsa package performs a systematic gene set analysis for identification of different types of gene set significance modules. The user can select the most appropriate analysis or combine them to provide insight into gene sets that respond in a similar manner to varying phenotypes and that might therefore be coregulated. For studies with more than two conditions, MAVTgsa not only provides the MANOVA test to identify gene sets consisting of differentially expressed genes but also implements three multiple comparison methods for post hoc analysis. The method implemented in MAVTgsa package has the advantage of real application. First, the MAVTgsa package provides the adjusted *P* values using both family-wise error rate (FWER) [25] and the FDR step-up procedure [26] using permutation method. Second, MAVTgsa is a permutation-based method to compute *P* values and adjusted *P* values. Third, MAVTgsa displays the results for individual genes test of significant gene sets. Finally, MAVTgsa draws the GST plot to display the empirical cumulative function for the ranked test statistics of a given gene set with the gene location and gene name above the plot. These allow the user to analyze the interesting gene set of the data easily. In addition, MAVTgsa provides a random forests-based procedure to identify gene sets in terms of predictive performance or in association with the continuous phenotypes. Random forests method has been proved to perform well in comparison with the other classification methods and successfully applied to various problems. Most importantly, it can accommodate multiclass and continuous phenotypes for the GSA, even if the associations between gene sets and phenotypes are nonlinear and involve complex high-order interaction effects.

## 6. Hard Ware and Software Specifications

The implementation and examples run of this package were conducted on a laptop computer with 2.8 GHz CPU and 3.0 GB RAM under the Microsoft Windows XP Professional SP3 using the R software version 2.14.1.

## 7. Availability 

The* MAVTgsa* package is available from http://cran.r-project.org/web/packages/MAVTgsa/.

## Figures and Tables

**Table 1:** The original *P* values and adjusted *P* values (FDR and FWE) of the OLS and Hotelling's *T* ^2^ test of P53 data.

GS name	GS size	OLS			*T* ^2^		
		*P* value	FDR	FWE	*P* value	FDR	FWE
hsp27Pathway	15	0∗	0	0	0.0001∗	0.0015	0.0003
p53hypoxiaPathway	20	0∗	0	0	0∗	0	0
p53Pathway	16	0∗	0	0	0∗	0	0
P53_UP	40	0∗	0	0	0∗	0	0
Radiation_sensitivity	26	0.0001∗	0.0062	0.0741	0∗	0	0
rasPathway	22	0.0015∗	0.0770	0.3618	0.0989	0.2621	0.0576
HTERT_DOWN	64	0.0029∗	0.1276	0.5490	0.0383	0.1636	0.1239
ST_Interleukin_4_Pathway	24	0.0079∗	0.3042	0.2743	0.0039∗	0.0375	0.1799
ck1Pathway	15	0.0089∗	0.3046	0.3107	0.0068∗	0.0582	0.1486
ngfPathway	19	0.0105	0.3102	0.2071	0.1313	0.2952	0.0862
inflamPathway	28	0.0133	0.3102	0.0737	0.0308	0.1492	0.0674
lairPathway	15	0.0134	0.3102	0.1119	0.0393	0.1636	0.2736
no2il12Pathway	17	0.0136	0.3102	0.2248	0.0245	0.1417	0.0549
cytokinePathway	21	0.0141	0.3102	0.2706	0.0290	0.1492	0.0217
badPathway	21	0.0157	0.3224	0.4702	0.0001∗	0.0015	0

*Denote *P* values < 0.01.

**Table 2:** Results of the MANOVA, 10-fold CV error, and post hoc test (Dunnett's method) of breast cancer data with three groups for nine pathways.

GS name	GS size	MANOVA *P*-value	10-fold CV	adjusted *P* value		Multiple comparisons	
			Error rate	FDR	FWE	*T*_2_ − *T*_1_	*T*_3_ − *T*_1_
androgen_receptor_signaling	72	0.0000∗	0.3438	0	0	0.2088	0.0027
apoptosis	187	0.0031∗	0.3542	0.0047	0.1751	0.5836	0.0517
cell_cycle_control	31	0.0000∗	0.3333	0	0	0.3823	0.0002
notch_delta_signalling	34	0.0040∗	0.3750	0.0051	0.2179	0.4558	0.0468
P53_signalling	33	0.0001∗	0.2917	0.0002	0.0078	0.4891	0.0029
ras_signalling	266	0.0049∗	0.3542	0.0055	0.5569	0.9132	0.0239
tgf_beta_signaling	82	0.0564	0.3438	0.0564	0.4885	0.6840	0.0664
tight_junction_signaling	326	0.0001∗	0.3854	0.0002	0.0294	0.2937	0.0058
wnt_signaling	176	0.0004∗	0.3542	0.0007	0.3299	0.6117	0.0037

*Denote *P* values < 0.01 in Wilks' Λ test.

**Table 3:** Type I error rate comparisons of two GSA methods for continuous phenotype, RF, and LCT, at a significance level of 0.05.

Method	*ρ* = 0.0	*ρ* = 0.3	*ρ* = 0.6	*ρ* = 0.9
*n* = 10, *p* = 20, *p*_1_ = 5				
RF	0.050	0.049	0.046	0.036
LCT	0.052	0.060	0.044	0.052

*n* = 20, *p* = 100, *p*_1_ = 20				
RF	0.051	0.042	0.049	0.052
LCT	0.060	0.051	0.050	0.064

*n* = 50, *p* = 200, *p*_1_ = 40				
RF	0.052	0.040	0.040	0.052
LCT	0.060	0.044	0.048	0.066
